# Vascular biomedicine in an era of chronic disease and multimorbidity

**DOI:** 10.1042/CS20180764

**Published:** 2019-05-14

**Authors:** Gemma Currie, Christian Delles

**Affiliations:** Institute of Cardiovascular and Medical Sciences, University of Glasgow, Glasgow, U.K.

**Keywords:** chronic disease, cardiovascular disease, multimorbidity, precision medicine, vascular biomedicine

## Abstract

It is increasingly common that patients present with more than one disease and that diseases are chronic in nature. Cardiovascular conditions such as hypertension, heart failure and stroke, renal diseases and cardiometabolic conditions such as diabetes are prime examples of chronic diseases which pose major challenges in contemporary healthcare provision. The complex features of multimorbidity call for precision medicine approaches that take comorbidity and chronicity into account. The research basis of chronic disease and multimorbidity, however, is currently in its infancy. This applies to all domains including basic, translational and clinical science. In this article we call for development of new models, smarter use of existing models and better characterisation of vascular and cardiovascular phenotypes in studies not directly related to cardiovascular diseases. This has the potential to further improve the quality of translational research, papers in journals such as *Clinical Science* and ultimately translate into better patient care.

## Introduction

**Figure 1:**
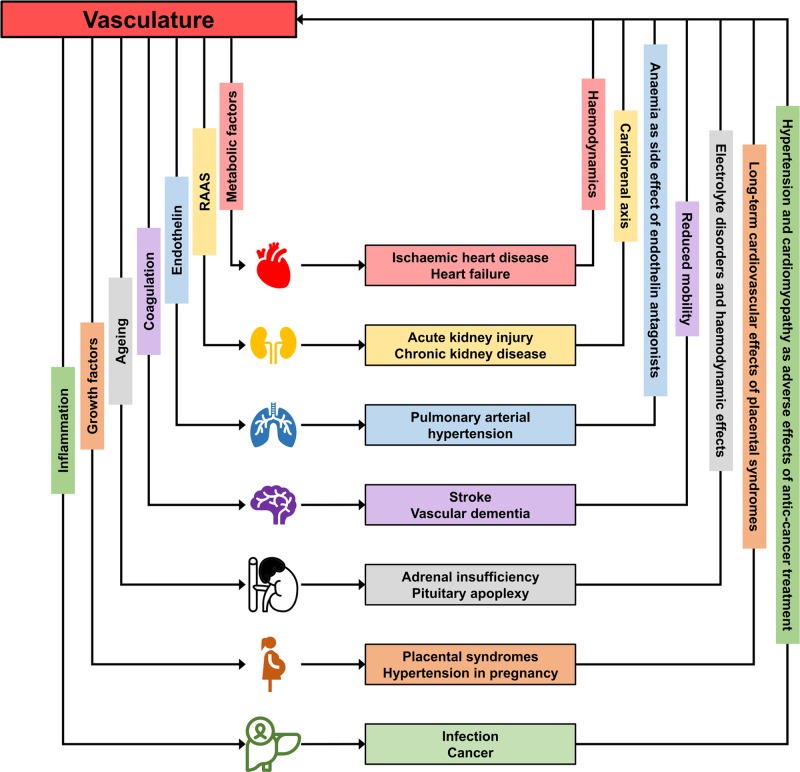
Interactions between the vasculature and specific organ systems The figure illustrates how the vasculature through various mechanisms affects organ systems and how these or organ-specific interventions in turn feed back to the vasculature. This is a schematic illustration and is by no means a complete or true representation of complex relationships but demonstrates the key role that the vasculature plays in the development of multi-organ disease. For example, through metabolic factors (lipids, diabetes) the vasculature affects cardiac function and the resulting haemodynamic changes in heart failure require vascular adaptations to maintain organ perfusion. Mechanisms such as inflammation, growth factors or the RAAS are other integrative features that play a role in multiple diseases. The allocation of only one factor to each organ in the figure is over-simplified but improves clarity. Some of the icons were made by Freepik from www.flaticon.com.

Cardiovascular diseases (CVD) remain the leading cause of morbidity and mortality worldwide [1]. It is often the vascular component of CVD that drives development of conditions such as myocardial infarction, heart failure and stroke; and whilst conditions such as aortic aneurysms, peripheral artery disease and various forms of vasculitis represent primary vascular conditions, the vasculature plays an important role in many seemingly non-vascular diseases including chronic renal disease and solid cancers (Figure 1). One of the major cardiovascular risk factors, hypertension, is considered a vascular disease, either originating from the vasculature or at least adversely affecting the vasculature where hypertension is due to other, for example endocrine, causes.

Such considerations have been repeatedly used to illustrate the central importance of vascular research, to attract funding and to improve the care of patients with CVD. In fact, over the last few decades we have witnessed major breakthroughs in our understanding of vascular physiology and pathophysiology. Examples include the discovery of the role of the renin–angiotensin–aldosterone system (RAAS) in vascular physiology; the discovery of endothelin and nitric oxide as endothelium-derived regulators of vascular tone and overarching principles such as oxidative stress and inflammation as key mediators of the development of vascular diseases. Many of these discoveries have translated into widely used treatments for vascular diseases including RAAS blocking agents for the treatment of hypertension [2]; NO donors for the treatment of angina [3] and endothelin antagonists for the treatment of pulmonary hypertension [4], and recent evidence suggests beneficial effects of anti-inflammatory strategies in the prevention and treatment of CVD [5].

Some of these pathophysiological principles are so universal and powerful that it is possible to measure them using traditional and often reductionist approaches that interrogate specific mechanisms, e.g. by genetically over or underexpressing key molecules within a given pathway or by pharmacological tools to stimulate or block receptors and other factors. However, neither do these principles exclusively affect a single (cardio)vascular phenotype nor do vascular diseases develop in isolation. It is well known to practicing clinicians that patients often present with more than one disease and that treating one condition can positively or negatively affect co-existing diseases.

## Multimorbidity and vascular diseases

The Academy of Medical Sciences in the United Kingdom has recently published their report ‘Multimorbidity: a priority for global health research’ to highlight the importance of this area from a research perspective [6]. Challenges are evident even in defining the concept of multimorbidity, where the report states that ‘inconsistent approaches to the definition and classification of multimorbidity have made the comparison and synthesis of findings from different research efforts challenging’. The WHO proposes a straightforward definition of multimorbidity as ‘being affected by two or more chronic health conditions in the same individual’ [7] and this definition can apply both to clinical and basic science research scenarios. Tightly linked with multimorbidity is the concept of chronic diseases, defined as pathologies that persist for more than 3 months, are not prevented by vaccines or cured by medication and are more common with ageing [8,9]. More than 75% of older adults have two or more co-existing chronic conditions, with CVD prevailing [10–12]. Multimorbidity is increasing in prevalence across all age groups, evolving into more of a clinical challenge than any single disease and is a key mediator of reduced life expectancy [12].

The clinical community has already realised the impact of multimorbidity and chronic disease on the future of health services and is in their daily clinical practice directly confronted with this challenge. In England the National Health Service (NHS) created the concept of a ‘House of Care’ to address the fact that ‘15 million people in England with long term conditions have the greatest healthcare needs of the population (50% of all GP appointments and 70% of all bed days) and their treatment and care absorbs 70% of acute and primary care budgets in England’ [13]. Strategies to transform clinical services such as ‘The Modern Outpatient: A Collaborative Approach 2017–2020’ by the Scottish Government are indeed driven by the experience that modern patient care has to address multiple and chronic conditions and cannot continue to work on single conditions in isolation [14].

Whilst the clinical challenges are imminent and already affect clinical practice, research into chronic diseases and multimorbidity is still in its infancy. This applies to all domains including basic, translational and clinical research. The key research question is that although some conditions may develop in parallel and independent from each other, it appears that there are patterns of comorbidities that cluster together either by deriving from similar pathogenetic principles or by causing each other; these clusters and their molecular origins are poorly understood.

For example, chronic kidney disease and heart failure are often of vascular origin, which has practical consequences for treatment with RAAS blocking agents and diuretics. Another example are chronic conditions such as rheumatoid arthritis or HIV infection which are often associated with metabolic changes, and where therapeutic approaches can affect metabolic health e.g. by alterations in lipid profiles. In another area of chronic diseases, cancer, treatment with chemotherapeutic drugs can cause cardiotoxicity, hypertension and other cardiovascular complications. Of course frailty that is associated with many chronic diseases can change cardiovascular risk profiles dramatically. Many of these disease and therapy-related consequences indeed affect the cardiovascular system, and the challenges are even greater in people with multimorbidity (Figure 1).

## Multimorbidity and precision medicine

Another major theme in life sciences and healthcare provision is precision medicine. Similar to developments in multimorbidity and chronic diseases, this concept originated from clinical practice where the aim to provide the best possible treatment to each individual patient has driven medicine for thousands of years. Concepts of precision medicine and multimorbidity share further common features in lack of a uniform definition and indeed, confusion with other terms that have been historically used to describe the same or related concepts such as ‘personalised medicine’ and ‘stratified medicine’ [15]. The National Research Council in the US have published a report ‘Toward Precision Medicine: Building a Knowledge Network for Biomedical Research and a New Taxonomy of Disease’ in 2011 [16]. The Council prefers the term ‘precision medicine’ where ‘the focus is on identifying which approaches will be effective for which patients based on genetic, environmental, and lifestyle factors’ [17] – clearly a research-oriented definition that has the potential to reshape the research landscape.

Precision medicine should indeed be viewed as a concept that sees the patient holistically and does not focus on single organ systems. Whilst most of the current successes of precision medicine are related to optimal therapeutic strategies in specific diseases, particularly in cancer, the concept is much broader and provides potential to study other organ systems that contribute to specific disease or are affected by specific disease and their therapies. The vasculature provides an opportunity to bring different organ systems together to one underlying shared factor (Figure 1) but there are other concepts such as inflammation and metabolic alterations that can be of equal integrative value.

In an analysis of potential cost-savings that could be achieved by implementation of precision medicine, Dzau et al. [18] demonstrated that the key benefits will derive from better treatment of CVD whereas other conditions such as cancer will benefit less in terms of cost savings – in part because of prevalence of disease, in part because such areas are a few steps ahead and already apply precision medicine principles such as targeted treatment based on a tumour’s genetic make-up. We propose that the real cost savings and improvement in health could be even more profound in CVD than projected by Dzau et al. if one takes multimorbidity into account. For example, avoiding cardiovascular side effects in the treatment of cancer or chronic lung diseases could further reduce treatment costs.

Chronic diseases and multimorbidity are in fact the most attractive areas for impactful implementation of precision medicine. Patients with long-term conditions urgently need treatments that can be tolerated for years and in many cases lifelong. Predicting the best possible treatment that causes the greatest benefit and avoids harm has transformative potential in the management of patients with chronic disease. This applies even more to those with multimorbidity where apart from ‘genetic, environmental and lifestyle factors’ [17], existing and potentially developing comorbidities are yet another factor that should inform precise therapeutic decisions. We already ask these questions in clinical care and often find that cardiovascular conditions feature prominently in our attempts to optimally treat people with multimorbidity: When do patients benefit from discontinuation of RAAS-blocking therapy when there is a conflict between deterioration of renal function and presumed cardiovascular protection? Do people with comorbidities that affect their overall survival benefit from the same blood pressure targets as those who are hypertensive but do not have other diseases? How do we choose antihypertensive or antidiabetic therapy to not only treat hypertension and diabetes but also positively modulate co-existing cardiovascular conditions such as heart failure? We do not want to suggest that clinicians are not aware of these questions or are not able to address them in current clinical practice. However, we suggest that precision medicine in general and research into precision medicine in particular should have a broader remit and include chronic disease and multimorbidity in all aspects from precision prevention to precision prediction, precision diagnostics and precision therapeutics.

It is interesting to note in this context that multimorbidity and precision medicine have stimulated discussions about repurposing drugs that have originally been developed for single specific conditions. Examples include the use of metformin in people with Type-1 diabetes to prevent CVD [19] and the cardiovascular protection and blood pressure lowering offered by SGLT-2 inhibitors [20]. Other lessons that have been learned in this context are that there is a lack of understanding even of the most basic physiological disease modifiers such as hormonal status, which is rarely assessed in clinical and experimental studies and will require more precise measurements than asking women about menopause.

## Translational research will become more complex

Reductionist approaches that study a single molecule or mechanism in a ‘clean’ model play an important role in dissecting specific aspects of pathophysiology. However, translation to other species, namely humans, and to people with complex (multifactorial) diseases and multimorbidity may not always be successful. Whilst the literature (including papers in this journal) is full of papers that describe the minutiae of disease mechanisms in well-controlled environments, the applicability of such findings to other models and ultimately to humans is limited due to different genetic and environmental background and presence of other conditions that may affect the proposed disease mechanism and interact with therapeutic approaches. This phenomenon also applies to clinical trials that are often characterised by highly selective inclusion and exclusion criteria that render them less readily applicable to ‘real-life’ patients with all their complexities and multimorbidities.

The experimental community has recently agreed on strategies to improve the quality of preclinical work and in particular how to address sex differences in the pathogenesis of disease [21]. Similar efforts are required to make preclinical studies fit for the clinical challenges related to chronic diseases and multimorbidity where holistic views are warranted. In the following section we will propose a number of steps that focus on the role of vascular diseases in the context of multimorbidity and chronic diseases.

## Vascular biomedicine and multimorbidity: moving forward

In Table 1 we propose four steps that apply to both basic and clinical research in the context of multimorbidity and chronic disease. We are concerned that the cardiovascular impact of novel therapies in non-cardiovascular conditions has not always been assessed but are convinced that the focus on multimorbidity will provide an opportunity to significantly improve the quality of our research and its potential to translate into clinical practice.

**Table 1:** Opportunities and challenges in preclinical and clinical vascular research in the context of multimorbidity

	Preclinical research	Clinical research
**Develop new research concepts**	Develop models of multimorbidity to understand common pathways	Develop new clinical trial concepts to include patients with multimorbidity and assess complex outcomes. Inclusion and exclusion criteria should reflect a more real-life population
**Utilise existing models to assess comorbidities**	Characterise existing models that display more than one phenotype to characterise comorbidities	Report cardiovascular outcomes in trials that are primarily conducted on non-cardiovascular conditions
**Utilise models of complex disease for cardiovascular research**	Utilise existing models of complex diseases, e.g. inbred strains with complex genetic background, with homology to human disease	Make use of ‘big data’ e.g. in the form of health record data and studies in primary care populations
**Assess cardiovascular phenotypes**	Assess vascular phenotypes in non-vascular studies and vice versa, assess non-vascular phenotypes in CVD models	Clinical trials should specifically assess vascular phenotypes and cardiovascular outcomes. Non-invasive tools and imaging technology will support this task

We appreciate that existing resources especially in the field of epidemiology and health record data can help us to understand the prevalence of disease clusters and how different diseases develop together; the cardiovascular system undoubtedly is one of the key systems affected by multiple pathways and therefore a natural focus of such research. We agree that such work should start immediately and is in fact already underway. However, we also strongly believe that there must be no delay in developing new concepts such as new preclinical models of chronic disease and new clinical trial designs to truly reflect multimorbidity.

Most importantly, we cannot any longer afford to ignore other systems in our research even if we are primarily interested in one specific disease, pathway or molecule. For example, a uromodulin knock-out model (Umod^−/−^) was originally described in 2004 by Bates et al. [22] in their studies into urinary tract infections; but it was not until 2014 that the blood pressure phenotype of this model was characterised [23]. However, comprehensive phenotyping of preclinical models is simply not possible for practical and financial reasons and larger initiatives are required to address this task and to standardise and quality-control phenotyping activities. In the United States initiatives such as the National Mouse Metabolic Phenotyping Centers [24] generate data beyond specific organ systems and thereby have the potential to support research into multimorbidity. In clinical research, comprehensive assessment of the phenome is delivered for example by the National Phenome Centre in the United Kingdom [25]. Whilst such large-scale programmes are probably the best way forward to tackle complex and multifactorial conditions we can all start at the local level and make sure that we look beyond our own horizon when designing preclinical and clinical experiments. Often minor changes in procedures will make tissue suitable for other research or generate data into organ systems that are not in the original focus and thereby support more holistic research. Ultimately, however, we will need to develop preclinical models that represent features of human ageing and multimorbidity better than currently existing models.

In clinical trials we have witnessed long-term adverse cardiovascular effects of thiazolidinediones, particularly of rosiglitazone, that were not discovered in the initial trials which focussed solely on their hypoglycaemic effect [26]. On a more positive note, the requirement to report cardiovascular outcomes in new trials into antidiabetic drugs has led to remarkable discoveries of beneficial effects particularly of SGLT-2 inhibitors [20].

Challenges that need to be overcome include seemingly simple issues such as a definition of multimorbidity in preclinical models in order to develop models that are representative of the situation in humans. Surgical manipulation such as 5/6 nephrectomy and aortic banding; pharmacological or dietary induction of disease for example by streptozotocin or feeding cholesterol-rich diets; and genetic models often generate acute conditions that are perfectly suitable to study specific pathophysiology but do not fully represent the slow development of multimorbidity with long-term interaction between affected organ systems. High phenotyping costs both preclinically and clinically are one of the practical hurdles that hinder progress in multimorbidity research. As indicated in Table 1, clinical trials offer opportunities beyond the primary research question and such aspects are already being explored as safety parameters, secondary or *post hoc* analyses. It is likely, however, that signals that explain interaction between organ systems and thereby the development of chronic disease and multimorbidity require novel tools including artificial intelligence approaches, comprehensive imaging and omics technologies.

## Conclusions

Policy makers and funding bodies recognise the challenges and opportunities related to multimorbidity and chronic diseases but it is the scientific community who need to drive this development forward. Descriptive studies in big data are important and already ongoing whereas basic research and clinical trials in multimorbidity are only slowly developing.

Vascular alterations are a common feature in many patients with chronic diseases and multimorbidity. Assessing and studying vascular phenotypes routinely in experimental models of any disease or disease cluster, as well as in human disease, is an obvious step towards understanding multimorbidity. We therefore invite basic and clinical researchers in non-cardiovascular areas of medicine to liaise with the cardiovascular community in order to develop study protocols that allow assessment of cardiovascular phenotypes. Depending on the specific area of research such strategies can include tissue harvesting, imaging studies and non-invasive functional studies.

Finally, we see a major role of scientific journals in promoting this field. *Clinical Science* is a journal with a scope of ‘Linking basic science to disease mechanisms’. This scope is deliberately broad and includes mechanisms of chronic disease and multimorbidity. In fact, we believe that the journal provides a particularly good home for quality papers in this exciting area of research.

## Abbreviations

CVD: cardiovascular diseases
RAAS: renin–angiotensin–aldosterone system
